# Improved efficacy of mitoxantrone in patients with castration-resistant prostate cancer after vaccination with GM-CSF-transduced allogeneic prostate cancer cells

**DOI:** 10.1080/2162402X.2015.1105431

**Published:** 2015-12-21

**Authors:** Joyce M. van Dodewaard-de Jong, Saskia JAM Santegoets, Peter M. van de Ven, Jurjen Versluis, Henk M. W. Verheul, Tanja D. de Gruijl, Winald R. Gerritsen, Alfons J. M. van den Eertwegh

**Affiliations:** a Department of Medical Oncology, VU University Medical Center, Amsterdam, the Netherlands; b Department of Clinical Oncology, Leiden University Medical Center, Leiden, the Netherlands; c Department of Epidemiology and Biostatistics, VU University Medical Center, Amsterdam, the Netherlands; d Department of Medical Oncology, Radboud University Medical Center, Nijmegen, the Netherlands

**Keywords:** Cancer vaccines, chemotherapy, docetaxel, GVAX, immunotherapy, mitoxantrone, prostate cancer

## Abstract

Previous vaccination studies in patients with castration-resistant prostate cancer (CRPC) showed improved survival without prolongation of progression-free survival (PFS). This might be explained by enhanced efficacy of subsequent therapies because of heightened immune status. We therefore evaluated the efficacy of chemotherapy in CRPC patients after immunotherapy. We retrospectively analyzed 28 patients who were treated with ipilimumab and GVAX, an allogeneic vaccine, and 21 patients who were randomized to GVAX or no vaccination. To study whether immune status was related to the efficacy of chemotherapy, frequencies of myeloid and lymphocyte subsets were determined. Of 28 patients treated with GVAX and ipilimumab, 23 patients received docetaxel and 13 patients mitoxantrone. Median PFS after docetaxel was 6.4 mo (range 0.8–11.2), while median PFS after mitoxantrone was markedly longer than expected (4.8 mo; range 1.4–13.7). High CD8^+^ICOS^+^ Tcell/Treg and pDC/mMDSC ratios were associated with relatively long PFS after mitoxantrone, suggesting a correlation between activated immune status and benefit of mitoxantrone. Analysis of 21 patients, randomized to GVAX or not, revealed a median PFS after docetaxel of 9.9 mo for vaccinated patients and 7.1 mo for unvaccinated patients. Interestingly, PFS after mitoxantrone (n = 14) was significantly longer in vaccinated patients as compared to controls (5.9 vs. 1.6 mo, *p* = 0.0048). In conclusion, mitoxantrone seems more effective in CRPC patients after immunotherapy, which may be related to the immune-stimulating effect of mitoxantrone in patients with heightened antitumor immunity. As this was a retrospective study with limited sample size, prospective studies are warranted to definitively show proof of principle.

## Abbreviations

APC: Allophycocyanin
BDCA: Blood dendritic cell antigen
BSA: Bovine serum albumin
Cdc: Conventional dendritic cell
CRPC: Castration-resistant prostate cancer
CTLA-4: Cytotoxic T-lymphocyte antigen 4
DC: Dendritic cell
DNA: DNA
ECOG: PS Eastern Cooperative Oncology Group Performance Score
Fab-M-FITC: Fragment antigen-binding—Mouse—Fluorescein isothiocyanate
FDA: Food and Drug Administration
FITC: Fluorescein isothiocyanate
FoxP3: Forkhead box P3
GM-CSF: Granulocyte-macrophage colony-stimulating factor
HLA-DR: Human leukocyte antigens-D related
HR: Hazard ratio
ICOS: Inducible T-cell costimulator
MDC: Macrophage-derived chemokine
MDSC: Myeloid-derived suppressor cells
PAP: Prostatic acid phosphatase
PBDC: Peripheral blood dendritic cell
PBMC: Peripheral blood mononuclear cell
PBS: Phosphate buffered saline
PCWG2: Prostate Cancer Working Group 2
pDC: Plasmacytoid dendritic cell
PE: Phycoerythrin
PerCP: Peridinin chlorophyll proteinCy5.5
PFS: Progression-free survival
PSA: Prostate-specific antigen
RECIST: Response Evaluation Criteria in Solid Tumors
Tact: Activated CD8^+^ T cells
Tcell: T cell
Th T: helper cell
Treg: Regulatory T cell
VUMC: VU University Medical Center

## Introduction

Prostate cancer is the most common malignancy in men in Europe and the United States.^1^ It is the fifth leading cause of cancer death in men worldwide, accounting for 7% (307.500) of all cancer deaths.^2^ Once metastatic disease occurs, first-line treatment consists of androgen blockade. However, after a median time of 18 to 24 mo, the cancer becomes castration resistant.^3^ In 2004, two phase-three trials showed that first-line treatment with docetaxel and prednisone improves survival of patients with CRPC and became subsequently standard of care.^4,5^ Before these publications, mitoxantrone was the only registered chemotherapeutic agent for men with CRPC because of its palliative benefit in approximately 30% of symptomatic patients. Yet, no improvement of overall survival was shown.^6,7^ After the introduction of docetaxel, mitoxantrone was considered to be a second-line option, because of its beneficial effect on quality of life and reasonable tolerability. The effect of crossing over from docetaxel to mitoxantrone has been evaluated in several studies. Median PFS from start of mitoxantrone as second-line treatment varied from 6.1 weeks to 3.4 mo.^8-13^

The introduction of the new agents enzalutamide and abiraterone, cabazitaxel and radium-223-chloride (all resulting in a survival benefit of several mo) has changed the therapeutic landscape of CRPC.^14-21^ However, despite all these new treatment options docetaxel chemotherapy remains an important cornerstone of the systemic treatment of CRPC. Mitoxantrone is currently less prescribed but still belongs to the treatment possibilities in advanced stage CRPC.

Over the past decade, different forms of immunotherapy have been developed and several clinical studies have demonstrated its potential benefit in patients with CRPC. Sipuleucel-T, a cancer vaccine consisting of activated autologous peripheral-blood mononuclear cells (PBMC's), received FDA-approval because of significant improvement of median overall survival as compared to placebo-treated patients.^22^ PROSTVAC-VF is another type of vaccine that is based on the use of two pox viral vectors encoding prostate-specific antigen (PSA) and three immune co-stimulatory molecules. Similarly to sipuleucel-T, increase of overall survival without effect on PFS was observed for PROSTVAC-VF-treated patients in a randomized phase II trial.^23^ The discrepancy between overall survival and PFS in these vaccination trials might indicate a relation between immunotherapy and response to subsequent therapies.

We performed a phase I dose-escalation trial to assess safety of combined prostate GVAX and ipilimumab immunotherapy in patients with metastatic CRPC.^24^ Prostate GVAX consists of a mixture of two allogeneic, hormone-sensitive (LNCaP) and hormone-resistant (PC-3) prostate cancer cell lines, which have been lethally irradiated and genetically modified to secrete GM-CSF. Ipilimumab is a monoclonal antibody against cytotoxic T-lymphocyte antigen 4 (CTLA-4), a co-inhibitory signal expressed on activated T cells. The CTLA-4 receptor on T-lymphocytes has a stronger binding affinity to B7 than CD28 and therefore serves as a dominant negative regulator of T-cell activation. In this phase I trial, we investigated the combination therapy of prostate GVAX and ipilimumab in chemotherapy naive CRPC patients and showed that it was tolerable and safe. In addition, we demonstrated several long-lasting partial PSA responses and relatively long survival, particularly in patients who developed a serological antitumor response and who showed a pre-treatment peripheral immune profile of low rates of immune suppressive regulatory T cells (Treg) and myeloid-derived suppressor cells (MDSC) combined with high rates of activated T helper (Th) cells and conventional dendritic cells (cDC).^24-26^

The observation that classical chemotherapeutic agents are also capable of stimulating tumor-specific immune responses has supported the rationale for combining vaccines with conventional therapies in patients with metastatic cancer.^27-31^ The observed survival benefit, without showing effect on PFS, could be explained by an improved efficacy of chemotherapy because of the induced antitumor immune response. To address this hypothesis we investigated the efficacy of subsequent chemotherapies in the combined GVAX and ipilimumab study.^24^ Because this analysis demonstrated a relatively long lasting response to mitoxantrone in vaccinated patients as compared to the results in literature, we subsequently investigated the efficacy of chemotherapy in patients who were randomly assigned to vaccination with prostate GVAX or no immunotherapy.

## Results

### Clinical results

Between Nov 18, 2004 and Dec 19, 2007 a total of 28 patients were included in the phase I prostate GVAX and ipilimumab study. Baseline characteristics at start of chemotherapy are summarized in Table 1. We identified 23 patients who received subsequent docetaxel and 13 patients who received mitoxantrone. Docetaxel was prescribed at a dosage of 75 mg/m^2^ intravenously every 3 weeks, whereas mitoxantrone was given at a dose of 12 mg/m^2^ intravenously 3 weekly. All patients that were treated with mitoxantrone had previously been treated with docetaxel except for one patient in whom the sequence of chemotherapy was reversed. Treatment with docetaxel or mitoxantrone was continued until progression occurred or side-effects became a limiting factor. The median number of docetaxel cycles was 8, ranging from 1 to 14 cycles (for one patient data were lacking due to treatment with docetaxel abroad). Patients  treated with mitoxantrone received a median number of 9.5 cycles (range 2–18). Median time between the last cycle of immunotherapy and start of docetaxel was 3.2 mo (range 0.6–27.3 mo). Mitoxantrone was started after a median of 16.1 mo (range 1.8–47.9 mo) following immunotherapy.

**Table 1.** Baseline characteristics at start chemotherapy of patients treated in the phase I dose-escalation trial studying the combination of ipilimumab with the whole-cell vaccine GVAX.

	Docetaxel	Mitoxantrone
	(n = 23)	(n = 13)
Mean age in years (range)	67.3 (45.5–80.9)	70.7 (55.5–83.0)
ECOG Performance Score		
- Not mentioned	0	1
− 0	4	3
− 1	16	6
− 2	2	3
− 3	1	0
Median PSA (µg/L)	298 (84–2633)	1286 (16–7861)
Median lactate dehydrogenase (U/L)	255 (162–840)	242 (198–808)
Median alkaline phosphatase (U/L)	298 (84–1395)	299(57–893)
Median hemoglobin (mmol/L)	7.1 (4.8–8.2)	7.0 (5.3–8.9)

Abbreviations: ECOG = Eastern Cooperative Oncology Group

A PSA decrease of ≥50% 12 weeks after start of therapy was reported in 8 out of 23 patients (35%) treated with docetaxel. Data of PSA levels were not available in 4 of 23 patients: three patients had progressive disease before week 12 and one patient was treated elsewhere. Five of the 13 patients receiving mitoxantrone (38%) had a more than 50% decline of PSA from baseline during chemotherapy. Twelve weeks after starting chemotherapy three of these PSA responses were still ongoing. In one of the patients treated with mitoxantrone, PSA follow up was missing because of clinical deterioration after two cycles of mitoxantrone.

Progression occurred in all 23 patients treated with docetaxel. Median time to progression from start of chemotherapy with docetaxel was 6.2 mo (95% CI: 3.5–8.9 mo; range 0.8–11.2 mo) and median overall survival was 16.1 mo (95% CI: 5.0–27.1). The 13 patients who received mitoxantrone had a median PFS of 4.8 mo (95% CI: 2.6–7.0 mo; range 1.4–13.7 mo) from the start of mitoxantrone therapy and median overall survival was 15.2 mo (95% CI: 11.1–19.3).

Because of the relatively long-lasting responses to chemotherapy in this retrospective analysis of patients previously treated with prostate GVAX/ipilimumab immunotherapy and the absence of a control group, we also analyzed time to progression after chemotherapy in patients who participated in the Vital 1 or Vital 2 studies.^32,33^ In total, 21 patients from our department participated in the Vital 1 (2 patients) or Vital 2 study (19 patients) and were treated with docetaxel. Fourteen of these patients were subsequently treated with mitoxantrone. Baseline characteristics at start of docetaxel and mitoxantrone are shown in. Table 2.

**Table 2.** Baseline characteristics of study subjects in the Vital 1 or Vital 2 study at start of chemotherapy.

	Docetaxel	GVAX^+^	GVAX^–^	Mitoxan-trone	GVAX^+^	GVAX^–^
	(n = 21)	(n = 9)	(n = 12)	(n = 14)	(n = 7)	(n = 7)
Mean age in years (range)	67.5	66	68.6	67.3	63.4	68.6
	(58.1–80.6)	(58.7–80.6)	(58.1–76.0)	(59.9–80.1)	(60.6–80.1)	(59.9–77.0)
ECOG PS						
- Not mentioned	2	1	1	1	0	1
- 0	6	3	3	2	1	1
- 1	11	4	7	8	4	4
- 2	2	1	1	2	1	1
- 3	0	0	0	1	1	0
Median PSA (µg/L)	184.9	184.9	220.9	332	376	173
	(5.7–3885.5)	(5.7–3885.5)	(14.9–3364.3)	(14–2822)	(14–1263)	(51–2822)
Median lactate dehydro- genase (U/L)	254	214	264	290	290	288
	(146–616)	(159–322)	(146–616)	(161–531)	(179–521) (2 missing)	(161–531)
Median alkaline phosphatase (U/L)	366	373	274	207	202	212
	(59–2413)	(59–1470)	(126–2413)	(79–1826)	(119–1826)	(79–790)
Median hemoglobin (mmol/L)	7.4	7.9	7.3	6.8	6.6	6.9
	(4.2–8.8)	(4.2–8.6)	(6.1–8.8)	(5.6–8.6)	(5.6–8.6)	(6.0–7.6)

Abbreviations: ECOG PS = Eastern Cooperative Oncology Group Performance Score, GVAX^+^ = Patients who were treated with GVAX, GVAX^–^ = Patients who received placebo-treatment

**Figure 1.**
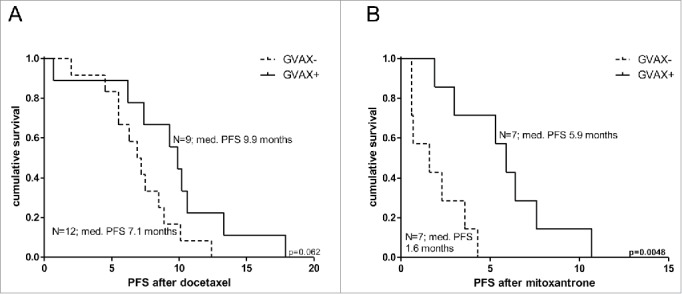
Kaplan–Meier curves of progression-free survival after docetaxel and mitoxantrone of study subjects from the VITAL1 or VITAL2 study. Kaplan–Meier curves of progression-free survival (PFS) after docetaxel (A) and mitoxantrone (B) treatment for subjects with (solid line) or without (dotted line) prior prostate GVAX treatment. Number of patients and corresponding median PFS for each group is given. Statistical significance of the survival distribution was analyzed by log-rank testing..

We compared response to chemotherapy between patients who were treated with prostate GVAX vaccination therapy in the Vital 1 or Vital 2 study and those who were not. Median PFS after docetaxel chemotherapy was not significantly different between patients that were vaccinated with prostate GVAX (n = 9) and patients that were not (n = 12). There was a trend for better response to docetaxel after vaccination with median PFS of 9.9 mo (range 0.7–17.9; 95% CI 5.37–8.43 mo) vs. 7.1 mo (range 2.0–12.4; 95% CI 8.15–11.65 mo) respectively, (log rank test *p* = 0.062; see also Fig. 1A). Interestingly, men who were previously treated with prostate GVAX experienced a significant prolonged PFS after mitoxantrone treatment compared with unvaccinated patients (median PFS 5.9 mo; range 1.9–10.7; 95% CI 4.36–7.44 mo vs. median PFS 1.6 mo; range 0.6–4.3; 95% CI 0.00–3.91 mo) respectively (log rank test *p* = 0.0048; see also. Fig. 1B)

### Immunological monitoring

**Figure 2.**
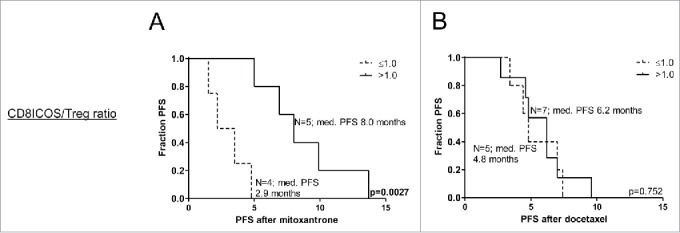
High Tact/Treg ratios after prostate GVAX/ipilimumab therapy are associated with significantly longer PFS following mitoxantrone. Kaplan–Meier curves of progression-free survival (PFS) following (A) mitoxantrone or (B) docetaxel treatment for prostate GVAX/ipilimumab-treated patients with high vs. low CD8^+^ICOS^+^/Treg ratios. Number of patients and corresponding median PFS for each group is given. Statistical significance of the survival distribution was analyzed by log-rank testing.

As evidence is accumulating that the immune status prior to treatment may determine clinical benefit of even conventional therapies such as chemotherapy, we assessed the immune status after immunotherapy in relation to subsequent responsiveness to chemotherapy. Frequencies of myeloid and lymphocyte subsets were determined after prostate GVAX/ipilimumab treatment. To this end, frequencies of activated CD8^+^ T cells (Tact), Tregs, Peripheral blood dendritic cells (PBDC) and mMDSC were determined, after which differences in DC/MDSC and Tact/Treg ratios between patients with a short or long PFS after docetaxel and mitoxantrone treatment were calculated. From a panel of activation markers, we observed an association between inducible T-cell costimulator (ICOS) expression levels on CD8^+^ T cells and survival after mitoxantrone treatment. Significantly higher CD8^+^ICOS^+^ T cell/Treg ratios were observed in patients with long PFS following mitoxantrone (*p* = 0.02), but not docetaxel treatment  (based on reported median PFS, see statistical paragraph in the Methods section). Similar results were observed in the myeloid compartment, with significantly higher pDC/mMDSC ratios for patients with long PFS following mitoxantrone (*p* = 0.0159), but not following docetaxel treatment (data not shown). Interestingly, CD8^+^ICOS^+^/Treg and pDC/mMDSC ratios of >1, i.e., more activated/stimulatory cells than suppressor cells, could be detected in the majority of patients that experienced long PFS after mitoxantrone treatment, whereas these ratios were ≤ 1 in all patients that experienced short PFS after mitoxantrone treatment. Patients who displayed these high CD8^+^ICOS^+^/Treg and pDC/mMDSC ratios had a significant longer PFS on mitoxantrone than patients who did not (*p* = 0.0027 and *p* = 0.0049 respectively). These significant associations were not observed for patients following docetaxel treatment, as demonstrated by Kaplan–Meier curves of PFS after mitoxantrone or docetaxel treatment with high vs. low CD8^+^ICOS^+^/Treg ratios in Fig. 2. These data strongly suggest that patients with a highly activated/more stimulatory immune profile prior to chemotherapy may be more susceptible to mitoxantrone treatment.

## Discussion

In this retrospective study, we describe the effect of chemotherapy (docetaxel and mitoxantrone) in patients who were previously treated with immunotherapy (ipilimumab and prostate GVAX) in a phase I dose-escalation trial. Treatment with docetaxel resulted in a median PFS of 6.2 mo which corresponds to the PFS of 6.3 mo reported in the SWOG 9916 trial (in which docetaxel was given in combination with estramustin), but is relatively shorter than the time to PSA progression of 7.7 mo reported in the TAX 327 study.^4,5^ However, definition of PSA progression in the latter differed from the current PCWG2 guidelines, because in responders PSA needed to rise ≥50% instead of ≥25% before PSA progression was established. Furthermore, we defined PFS as a composite endpoint of PSA progression, radiological progression or death.

Interestingly, the patients who were treated with mitoxantrone had a relatively higher response rate (38%) and a longer median PFS (4.8 mo) than has been reported in previous studies (RR 6–18%; PFS 6.1 weeks–3.4 mo).^8-12^ Because this analysis lacked a control group, it cannot be excluded that this observation might be related to patient selection and/or differences in study design. Therefore, we subsequently investigated the efficacy of chemotherapy in patients who were randomly assigned to vaccination-therapy or not in the Vital 1 and Vital 2 study. PFS after docetaxel was comparable in vaccinated and unvaccinated patients. Interestingly, vaccinated patients who were treated with mitoxantrone had a significantly longer PFS as compared to the non-vaccinated group (Fig. 1B), which confirms the results of our first analysis. These results support the hypothesis that response to chemotherapy, in particular mitoxantrone, might be improved when administered subsequent to immunotherapy. The observed prolonged PFS after chemotherapy in our study population might be related to the immuno-modulatory effect of chemotherapy.

In our study population, especially mitoxantrone chemotherapy resulted in better responses and PFS than previously reported in literature. Mitoxantrone is a DNA-reactive agent closely related to the anthracyclins, which intercalate into DNA, thereby causing cross-links and strand breaks. Besides the direct cytotoxic effect on tumor cells, anthracyclins may also stimulate the host immune system to attack cancer cells. Following mitoxantrone treatment, cancer cells become apoptotic and release specific antigens which are presented by dendritic cells.^34^ Dendritic cells have the crucial ability to cross-present tumor-associated antigens and to cross-prime cytotoxic T cells.^34^ Consequently, cancer cells that succumb to the lethal effect of mitoxantrone, serve as a therapeutic vaccine and stimulate the host immune system to attack other tumor cells. This process is called immunogenic cell death and was reported for mitoxantrone and other closely related anthracyclins, but importantly, not for docetaxel.^28-30,34-37^ This is in line with the observations in our phase I study population, showing no significant differences in responses and PFS between the GVAX vaccinated and unvaccinated patients after docetaxel treatment. Beside the immunogenic effects on tumor cells, mitoxantrone can also promote rapid dendritic cell differentiation which may support the development of a strong immune response as well.^38^ In an experimental murine tumor model, it was demonstrated that doxorubicin enhanced the tumor-specific CD8^+^ T cell response that was important for the therapeutic efficacy of this anthracyclin.^39^ We have recently reported increased frequencies of activated T cells as well as activated dendritic cells upon treatment with prostate GVAX and ipilimumab.^25^ To investigate whether an activated immune status post-immunotherapy was associated with improved efficacy of mitoxantrone, we did an exploratory immunological analysis in a small number of patients at follow-up after treatment with GVAX and ipilimumab and prior to treatment with subsequent chemotherapy. Interestingly, we observed a more stimulatory immune profile in the patients with a longer PFS after mitoxantrone treatment than in the patients with a short PFS. Our finding of a favorable immune status possibly predicting clinical benefit on mitoxantrone but not docetaxel is in line with the immunogenic cell death inducing ability of the former, and the inability of the latter. The capacity of the immune system to respond upon mitoxantrone-induced immunogenic cell death may in large part contribute to clinical benefit, as previously suggested by Zitvogel et al.^40^ In light of this assertion, it stands to reason that this ability should be improved after immuno-stimulatory therapy and argues in favor of immunotherapy preceding mitoxantrone treatment, as supported by our data. Obviously, this hypothesis was tested in a very small subgroup and should be substantiated in larger future studies. However, there is no evidence that docetaxel can induce immunogenic cell death.^30^ This is in line with the observations in our study population, showing no significant differences in responses and PFS after docetaxel in vaccinated as compared to unvaccinated patients.

This retrospective analysis has several intrinsic limitations. First, the number of patients in this analysis is relatively small. It is possible that the differences we found in PFS between vaccinated and non-vaccinated patients are caused by confounding factors such as PSA level, Gleason score, previous treatments and time between vaccination and start of chemotherapy. However, due to the small sample size it was not possible to perform a multivariate analysis to correct for possible confounders. Nevertheless, this is the first study in which the results of chemotherapy with docetaxel and mitoxantrone after immunotherapy with GVAX and ipilimumab or GVAX alone are described.

Second, this analysis had a retrospective study design. Therefore, starting and stopping of chemotherapy with docetaxel and mitoxantrone was not performed according to a study protocol but to the best insight of the treating oncologist. The absence of clear inclusion criteria before start of chemotherapy can result in serious heterogeneity of the study population. Docetaxel was the preferred treatment for almost all patients after vaccination therapy, but mitoxantrone was started at different time points in the course of the disease. In addition, there were no pre-specified evaluation moments after start of chemotherapy, which could influence the outcome measure PFS. However, PSA values were frequently measured in almost all patients. We chose PFS instead of overall survival as primary outcome measure in this retrospective study population because treatment courses after chemotherapy were very different between patients. Therapeutic regimens after docetaxel and mitoxantrone for example consisted of carboplatin/docetaxel, samarium-153-EDTMP, abiraterone/prednisone, cabazitaxel and ketoconazol/hydrocortisone in various sequences and frequencies. Using PFS as endpoint enabled us to study the antitumor effect of mitoxantrone alone without these confounding factors.

For our comparative analysis of vaccinated vs. unvaccinated patients, we collected data from patients who participated in the Vital 1 or Vital 2 study.^32,33^ Unfortunately, both studies were terminated before planned accrual was completed. The Vital 2 study was terminated after an interim analysis demonstrated a shorter median survival (12.2 mo vs. 14.1 mo) in the group treated with both GVAX and chemotherapy compared to the group treated with docetaxel/prednisone. Failure of the Vital 2 study could be due to the fact that prednisone (having a proven antitumor effect) was only administered in the control arm. After this, an unplanned futility analysis of the Vital 1 study was performed which indicated that it was unlikely to achieve the primary endpoint (overall survival) of the trial. In summary, neither one of these phase III trials demonstrated a survival benefit of vaccination therapy with GVAX. However, based on the working mechanism of immunotherapy, this could be due to the relatively short follow-up in both trials. The phase three trials with sipuleucel-T in CRPC and ipilimumab in metastatic melanoma showed that longer follow up is essential to reveal a survival benefit, which could be related to the enhanced efficacy of subsequent therapies as observed in our study.^22,41,42^

In conclusion, we described the effect of chemotherapy with docetaxel and mitoxantrone in patients with metastatic CRPC who were previously treated with immunotherapy (consisting of prostate GVAX with or without ipilimumab). Although sample sizes were small, our data suggest that metastatic CRPC patients previously treated with immunotherapy experience prolonged PFS following mitoxantrone treatment. To further substantiate our findings, it would be interesting to analyze the response to chemotherapy of patients with CRPC who have been treated in large trials studying any form of immunotherapy (e.g., sipuleucel-T, PROSTVAC-VF and ipilimumab). Moreover, prospective randomized controlled trials are warranted to further examine the possible synergism between mitoxantrone and immunotherapy.

## Patients and methods

This study (ClinicalTrials.gov, number NCT01510288) was a phase I dose-escalation trial in which 28 patients were enrolled who had metastatic CRPC and had not been previously treated with chemotherapy.^24^ All patients received a 5 × 10^8^ cell priming dose of GVAX intradermally on day 1 with subsequent intradermal injections of 3 × 10^8^ cells every 2 weeks for 24 weeks. The vaccinations were combined with intravenous ipilimumab every 4 weeks. Twelve patients were enrolled in the dose-escalation cohort (cohorts of three patients; each cohort received an escalating dose of ipilimumab at 0.3, 1.0, 3.0 or 5.0 mg/kg). Subsequently, 16 CRPC patients were treated at dose level 3.0 mg/kg. All patients provided written informed consent and the study was approved by the local ethics review board.

After completing the study or in case of progressive disease, patients were referred to their clinician for further treatment. We performed a retrospective study on all patients. We collected data about further treatment schedules (kind of therapy, number of cycles received), serial levels of serum PSA, radiological follow-up and data of death. Descriptive statistics were used to summarize patient characteristics by treatment group. Progression was defined as a composite endpoint of PSA progression (defined as PSA increase of ≥25% and ≥2 ng/mL above the nadir, confirmed by a second value three or more weeks later), objective progression on imaging (using RECIST 1.1) or clinical progression as judged by the treating physician or death).

Once we collected data of all patients in the phase I trial, we decided to extend our retrospective analysis with patients from the VU University Medical Center (VUMC) who participated in either the Vital 1 study or the Vital 2 study. The Vital 1 study (ClinicalTrials.gov, number NCT00089856) was a randomized phase III study of prostate GVAX vs. docetaxel and prednisone in patients with CRPC who were chemotherapy naive.^32^ Based on a futility analysis showing <30% chance of meeting the primary endpoint this study was terminated in 2008. At the VUMC, two patients were randomized to prostate GVAX and none to the docetaxel arm. The Vital 2 study (ClinicalTrials.gov, number NCT00133224) was a randomized phase III study of prostate GVAX and docetaxel vs. docetaxel/prednisone in symptomatic chemotherapy naive CRPC patients.^33^ This study was terminated due to an independent data monitoring committee recommendation. At the VUMC, seven patients were randomized to prostate GVAX and docetaxel and 12 to docetaxel/prednisone. We combined data from the Vital 1 and Vital 2 study and compared PFS after chemotherapy in 9 vaccinated patients vs. 12 unvaccinated patients.

### Blood sampling for immunological monitoring

For immunological monitoring, blood samples were taken from the prostate GVAX/ipilimumab-treated patients at follow-up, which was 4 weeks after the last prostate GVAX injection and 8 weeks after the last ipilimumab administration. Blood samples of 14 patients out of the 23 who received docetaxel after GVAX/ipilimumab were available for analysis, whereas blood samples of 11 out of 13 patients who were treated with mitoxantrone could be analyzed. PBMC's were isolated by density centrifugation (Nycomed AS, Oslo, Norway), after which they were either immediately stained and assessed by flow cytometry or cryopreserved for later analysis.

### Antibodies and 4-color flow cytometry

Circulating myeloid and lymphocyte subsets were assessed by routine PBMC flow cytometry analysis as described earlier.^25^ Cell surface antibody staining was performed in PBS/0.1% BSA/0.02% Sodium-Azide for 30 min at 4°C. Intracellular FoxP3 staining was conducted with the antihuman FoxP3 staining kit (eBioscience, catalog number 77–5774–40), according to manufacturers' protocol. The following antibodies were used (catalog numbers within brackets): fluorescein isothiocyanate (FITC)-, phycoerythrin (PE)-, peridinin chlorophyll protein-Cy5.5 (PerCP)- or allophycocyanin (APC)-labeled antibodies directed against human CD3 PerCP (332772), CD3 PE (345765), CD3 FITC (345763), CD4^+^ FITC (345768), CD8^+^ PerCP (345774), CD11c APC (333144), CD14 PerCP Cy5.5 (550787), CD123 PE (555644) CD16 FITC (555406), CD19 FITC (555412), CD19 PE (345789) CD56 FITC (345811), CD25 APC (340907), HLA-DR FITC (347400), HLA-DR APC (347403, all BD Bioscience), FoxP3 PE (12–4776–42), ICOS-biotin (13–9948–82), IgG1 biotin (13–4714–85), APC-conjugated streptavidin (17–4317–82, all eBioscience, San Diego), Fab-M-FITC (1022–02, Southern Biotec, Birmingham, AL) and blood dendritic cell (DC) antigens BDCA1 FITC (130–090–507), BDCA2 FITC (130–090–510), BDCA3 FITC (130–090–513, all from Milteny Biotec, Bergisch Gladbach, Germany) and MDC8 (a kind gift from Dr. Rieber) and matching isotype control antibodies IgG1 FITC (345815), IgG1 PE (345816), IgG1 PerCP Cy5.5 (550795), and IgG1 APC (345818, all BD Bioscience). Stained cells were analyzed on a FACScalibur (BD Biosciences) using Cell Quest software. Events collected were 100,000–150,000 per sample.

### Myeloid and lymphocyte subset definitions

PBDC frequencies were determined on the basis of expression of BDCA markers: DC belonging to the so-called conventional DC1 (cDC1) subsets were identified as CD11c^high^CD19^−^CD14^−^BDCA-1/CD1c^+^, and plasmacytoid DC (pDC) were detected as CD11c^−^CD14^−^CD123^high^BDCA-2^+^.^43^ Monocytoid MDSCs were defined as Lin^−^CD14^+^HLA-DR^neg/low^ cells.^44^ Tact were defined as CD8^+^ICOS^+^ cells and regulatory T cells (Tregs) as CD3^+^CD4^+^CD25^high^ and FoxP3^+^. Activated T cell/Treg (Tact/Treg) ratio was determined by dividing frequency of CD8^+^ICOS^+^ T cells by frequency of CD4^+^CD25^high^FoxP3^+^ Tregs. Similarly, DC/MDSC ratios were determined by dividing frequency of cDC1 or pDC by the frequency of mMDSC.

### Statistical analyses

The Kaplan–Meier method was used to estimate median time to progression from start of chemotherapy with docetaxel and from subsequent start of chemotherapy with mitoxantrone. The log-rank test was used to compare PFS and survival between vaccinated and unvaccinated patients from the Vital 1 and Vital 2 trials.

To determine whether the immune status of patients prior to chemotherapy treatment impacted their responsiveness to chemotherapy,  differences in DC/MDSC and Tact/Treg ratios between patients with a good or poor response after docetaxel or mitoxantrone treatment were analyzed with the two-sample Mann–Whitney U test. To this end, patients were grouped into two groups, i.e., short vs. long PFS after docetaxel and mitoxantrone treatment. The cut-offs for short/long PFS were based on the previously described median PFS after docetaxel and mitoxantrone treatment in similar patient populations, i.e., 6.3 mo for docetaxel-treated and 3.5 mo for mitoxantrone-treated patients.^4,10,11^

To determine whether the cDC1/mMDSC, pDC/mMDSC and Tact/Treg ratios were useful for PFS prediction after docetaxel of mitoxantrone treatment, patients were grouped based on a DC/MDSC or Tact/Treg ratio >1 (i.e., more activated/stimulatory cells than suppressor cells) or ≤ 1 (i.e., equal amounts or less activated/stimulatory cells than suppressor cells), after which PFS for the two groups was plotted using the Kaplan–Meier method.

All statistical analyses were performed with either GraphPad or SPSS software. Differences were considered significant when *p* < 0.05.
